# Remote Interference Discrimination Testbed Employing AI Ensemble Algorithms for 6G TDD Networks

**DOI:** 10.3390/s23042264

**Published:** 2023-02-17

**Authors:** Hanzhong Zhang, Ting Zhou, Tianheng Xu, Honglin Hu

**Affiliations:** 1 Shanghai Advanced Research Institute, Chinese Academy of Sciences, Shanghai 201210, China; 2 School of Information Science and Technology, ShanghaiTech University, Shanghai 201210, China; 3 School of Electronic, Electrical and Communication Engineering, University of Chinese Academy of Sciences, Beijing 100049, China; 4 School of Microelectronics, Shanghai University, Shanghai 200444, China; 5 Shanghai Frontier Innovation Research Institute, Shanghai 201100, China

**Keywords:** remote interference, interference discrimination testbed, ensemble algorithms, Bagging

## Abstract

The Internet-of-Things (IoT) massive access is a significant scenario for sixth-generation (6G) communications. However, low-power IoT devices easily suffer from remote interference caused by the atmospheric duct under the 6G time-division duplex (TDD) mode. It causes distant downlink wireless signals to propagate beyond the designed protection distance and interfere with local uplink signals, leading to a large outage probability. In this paper, a remote interference discrimination testbed is originally proposed to detect interference, which supports the comparison of different types of algorithms on the testbed. Specifically, 5,520,000 TDD network-side data collected by real sensors are used to validate the interference discrimination capabilities of nine promising AI algorithms. Moreover, a consistent comparison of the testbed shows that the ensemble algorithm achieves an average accuracy of 12% higher than the single model algorithm.

## 1. Introduction

Massive access is defined as a typical scenario of sixth-generation (6G) communications by IMT-2030 Promotion Group. Numerous Internet of Things (IoT) devices will be connected to the communication network [1]. However, the remote interference caused by the atmospheric duct brings about the interference signal exceeding the guard period (GP), which interferes with the co-frequency uplink signal reception of low-power IoT devices in 6G time-division duplex (TDD) networks and increases the risk of communication interruption for mobile users.

**Figure 1:**
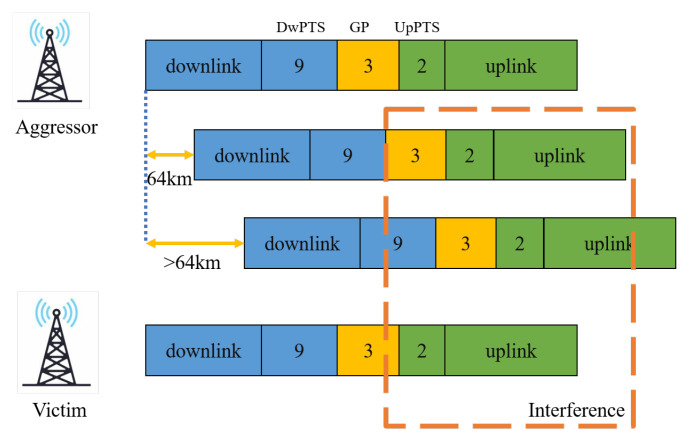
Remote interference in TDD system, in which GP is the guard period and PTS is the pilot time slot.

The TDD mode, which prominently suffers from the interference of the atmospheric duct, refers to the uplink and downlink utilizing the same frequency band to transmit information at different times [2]. The GP, as shown in Figure 1, is applied to protect the uplink signal from the interference of the downlink signal [3]. The interference signal can be filtered by the sensor within the GP protection range. However, the distance of remote interference will far exceed this range. The atmospheric duct, which results from non-standard meteorological conditions, captures the electromagnetic wave and induces the signal to propagate in the ducting layer [4]. The atmospheric duct captures the signal and allows the signal to propagate beyond the GP maximum protection distance with low path loss [5]. Thus, the captured signal maintains a high signal strength and interferes with the uplink signal reception of remote IoT devices [6].

According to statistics, China, Japan, Netherlands, and the United States have suffered from the interference of the atmospheric duct for a long time [7,8,9,10]. In the process of 5G research, remote interference has attracted the attention of researchers. 3GPP promoted a remote interference project in the standardization research of 5G-Beyond to analyze the adverse impact of the remote interference on communication systems [11]. Moreover, ITU recommended that the atmospheric duct should be considered in channel modeling [12]. Ericsson and other telecom companies began monitoring and analyzing the effects of remote interference on communication systems [13]. Clearly, the negative effects of the atmospheric duct have attracted the attention of many researchers. In the future research of 6G communications, remote interference will also be an unavoidable problem.

Currently, there are two main methods used to detect and estimate the atmospheric duct: (1) using meteorological factors to calculate the atmospheric refractive index [14]; (2) using radar to measure the atmospheric duct [15]. Nonetheless, Method (1) usually utilizes PETOOL platform to generate simulation data [16]. It contains several assumptions to generate simulation data, which hardly reflect reality. Method (2) is suitable for ocean scenarios and is expensive. In addition, remote interference typically occurs on land where water vapor evaporation is large. Considering complex scenarios and large amounts of data, traditional modeling methods do not work.

Motivated by the above challenges, a remote interference discrimination testbed employing AI ensemble algorithms for 6G wireless communications is proposed. The contributions of this paper are summarized as follows:

- A remote interference discrimination testbed is originally proposed, which adopts 5,520,000 TDD network-side interfered data to discriminate the remote interference. A large number of measurement data could effectively appraise the interference discrimination ability of different AI algorithms;
- The testbed verifies the interference discrimination ability of two types of a total of nine AI algorithms, which lays the foundation for the application of the testbed in different hardware environments;
- According to the consistent comparison, numerical results illustrate that the ensemble algorithm achieves an average accuracy of 12% higher than the single model algorithm. The work fills the gap of remote interference in the 6G communication scenario and helps mobile operators improve network optimization capabilities under remote interference.

The remainder of the paper is organized as follows. In the next section, the recent studies of atmospheric duct and the framework of the proposed testbed are introduced. Section 3 shows the employed ensemble discriminant algorithms. Extensive experiments are presented in Section 4. Finally, the conclusions are summarized in Section 5.

## 2. Related Work and Testbed Design

### 2.1. Related Work

While most of the existing research literature on the atmospheric duct has focused on calculating the height of the ducting layer, there has been little analysis of the interference discrimination in communication systems. Currently, there are two main approaches to detect and estimate the atmospheric duct, including theoretical calculations and practical measurements.

Ray-optics (RO) method and parabolic equation (PE) method are developed to calculate the trajectory of the ducting layer. For example, a RO method was applied to calculate ray trajectories with atmospheric ducts in Ref. [17]. The authors analyzed delay spreads to determine the fading behavior of the channel, which compensated for a realistic analysis for the delay spread of ducting channels. A PE-based tool (PETOOL) was developed in Ref. [18], who analyzed the ideal ducting effect from 800 MHz to 20 GHz.

Considering the interference of the duct on the electromagnetic wave signal, some studies utilized radar and other equipment for measurement. In Ref. [19], a comprehensive observation experiment was carried out in the Guangdong Province of China. A shore-based navigation radar was used for over-the-horizon detection and radiosondes were used to measure the atmospheric profile. A method of detecting atmospheric ducts using a wind profiler radar and a radio acoustic sounding system was proposed in Ref. [20]. The measurements were carried out in the Liaoning Province of China. These activities all take place at sea, and the expensive cost and restrictions hinder land measurement.

### 2.2. Testbed Design

**Figure 2:**
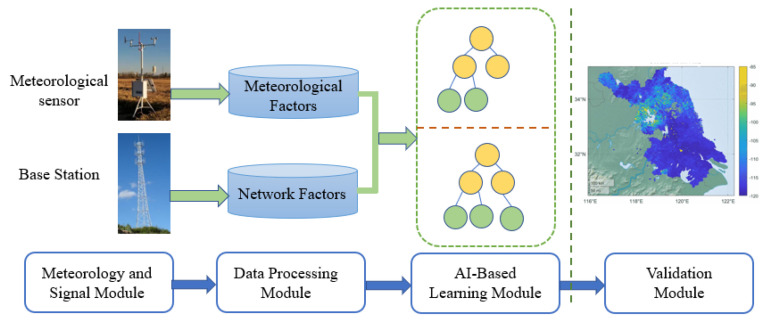
The framework of the testbed.

The proposed remote interference discrimination testbed is shown in Figure 2. It consists of four modules, including meteorology and signal module, data processing module, AI-based learning module, and validation module.

First of all, the meteorology and signal module adopts sensors to collect meteorological and network-side data. Secondly, in the data processing module, the collected data is cleaned and divided into two parts: meteorological factors and network factors. Then, the factors are input into AI-based learning module to acquire data characteristics. Finally, the validation module uses the measurement data to verify the interference discrimination ability of the model. Our previous work has completed the meteorology and signal module, and validation module [21]. In the following, we focus on introducing the data processing module and AI-based learning module.

Without loss of generality, a channel with atmospheric duct interference is considered. In data processing, interference discrimination requires elucidating which factors are relevant for the wireless channel under ducting interference. The contributory factors are deduced in the following, which consists of meteorological factors and network factors.

#### 2.2.1. Meteorological Factors

Atmospheric refraction is the bending of electromagnetic waves propagating in the atmospheric media. The degree of refraction could be described by the refractive index, which is expressed as [17]

(1) $$n=\frac{c}{v},$$

where *c* represents the light speed, and *v* refers to the velocity of the electromagnetic wave in the medium. The atmospheric refractivity is employed to replace the refractive index due to the minuscule value of *n* being ignored when calculated for most cases [22]. The refractivity can be described as [12]

(2) $$N=\left(n-1\right)\times 10^{6}=\frac{77.6}{T}\times \left(p+\frac{4810e}{T}\right),$$

where *T* denotes the temperature, *p* represents the atmospheric pressure, and *e* indicates the vapor pressure.

Notably, the curvature of the earth needs to be considered since the signal captured by the atmospheric duct is capable of traveling long distances. As a result, the modified refractivity, which considers the curvature of the earth, can be expressed as [12]

(3) $$M=N+\frac{h}{r_{e}}\times 10^{6},$$

where *h* denotes the height above ground, and $r_{e}$ is the earth radius. The atmospheric duct occurs when $\frac{dM}{dh}<0$. The appearance of the atmospheric duct is related to meteorological parameters, whose changes are inseparable from time.

#### 2.2.2. Network Factors

The PE method, utilizing paraxial approximation of the Helmholtz equation, could model the changes of refractivity in the atmosphere and simulate complex boundary conditions. As such, the PE-based path loss model, which integrates diverse conditions well, can be represented as [23]

(4) $$L_{p}\left(z,h\right)=-20\mathrm{lg}\left|u\left(z,h\right)\right|+20\mathrm{lg}\left(4\pi \right)+10\mathrm{lg}\left(z\right)-30\mathrm{lg}\left(\lambda \right),$$

where $L_{p}$ denotes the path loss of the signal, *z* represents the horizontal distance of signal propagation, λ is the carrier wavelength, and *u* refers to the field strength, which can be written as [23]

(5) $$u=\sqrt{2\pi }\int_{-\infty }^{+\infty }B\left(\theta \right)e^{2inp_{b}h}dp_{b},$$

(6) $$sin\left(\theta \right)=\lambda p_{b},$$

where *B* refers to the beam function, θ denotes the down tilt angle, and $p_{b}$ indicates the beam. When the antenna is modeled as a Gaussian function, *B* can be formulated as [23]

(7) $$B\left(\theta \right)=Ae^{\left(-2\mathrm{lg}2\frac{\theta^{2}}{\beta^{2}}\right)},$$

where *A* denotes the normalization constant, and β refers to the half-power beamwidth.

Under these circumstances, the initial field strength can be written as [23]

(8) $$u\left(0,h\right)=A\frac{k\beta }{2\sqrt{2\pi \mathrm{lg}2}}e^{-ik\theta h}e^{-\frac{\beta^{2}}{8\mathrm{lg}2}k^{2}{\left(h-h_{a}\right)}^{2}},$$

where *k* indicates the incident wave beam, and $h_{a}$ represents the antenna height.

The solution of the field strength can be described as [23]

(9) $$\frac{\partial u}{\partial h}\left(z,h\right)=ik\left(\sqrt{\frac{1}{k^{2}}\frac{\partial^{2}}{\partial h^{2}}+n^{2}{\left(1+\frac{h}{r_{e}}\right)}^{2}-1}\right)u\left(z,h\right).$$

Equation (9) needs to be solved by the Fourier transform and inverse transform. The relationship between field strengths can be expressed as [23]

(10) $$U\left(z,p_{b}\right)=\int_{-\infty }^{+\infty }u\left(z,h\right)e^{-2i\pi p_{b}h}dh,$$

(11) $$u\left(z,h\right)=\int_{-\infty }^{+\infty }U\left(z,p_{b}\right)e^{2i\pi p_{b}h}dp_{b}.$$

After finishing the Fourier transform, the increment can be calculated as [23]

(12) $$u\left(z+\Delta z,h\right)=e^{ik\Delta z}\left(\sqrt{\frac{1}{k^{2}}\frac{\partial^{2}}{\partial h^{2}}+n^{2}{\left(1+\frac{h}{r_{e}}\right)}^{2}-1}\right)u\left(z,h\right).$$

As can be seen from the above analysis, the PE method adopts the split-step Fourier transform to solve the equation due to the complex nonlinear relationship between the path loss of the signal and contributory factors. In summary, the contributory factors of the atmospheric duct include temperature, atmospheric pressure, relative humidity, time, longitude, latitude, antenna height, and down tilt angle. These factors mentioned above affect the path loss of the signal.

Considering the contributory factors, the corresponding data is selected from the dataset. Traditional modeling methods struggle to effectively learn and represent data features in the presence of huge amounts of data, so AI-based learning methods have emerged as a promising solution.

## 3. AI-Based Discriminant Algorithms

The processed data is input to the AI-based learning module to generate the feature model. The model can be adopted to discriminate the remote interference and warn the operator to operate to avoid remote interference. Obviously, an accurate model is crucial for the interference discrimination framework. The discriminant algorithm is mainly separated into two parts, including the single model algorithms, and the ensemble algorithms [24]. The details of the discriminant algorithms are as follows.

### 3.1. Single Model Algorithms

The single model algorithms have been applied in many fields. Some investigations have verified that some single model algorithms have pleasant performance in remote interference discrimination, which is the focus of the subsection.

Most single model algorithms adopt mathematical expressions to judge categories. For example, nearest distance matching, distribution model matching, and so on. Single model algorithms often achieve satisfactory performance in communication problems such as low interference channel estimation [25]. The channel contributory factors of interference discrimination exist as complex nonlinear relationships, and require a high demand for single model algorithms. The single model algorithms, which have been employed for interference discrimination, will be introduced as follows [26].

#### 3.1.1. kNN

The k-Nearest Neighbors (kNN) algorithm is an earlier supervised machine learning algorithm. The keystone of kNN is using *k* adjacent values to represent sample points [27]. The category of sample points is determined by the *k* nearest neighbors, which is the same as the majority of the neighbors. Many ways can be applied to express the distance between points, including the Euclidean distance, Manhattan distance, cosine distance, Chebyshev distance, and so forth [28].

The Euclidean distance is often selected as the calculation index, which can be expressed as [28]

(13) $$d_{\mathrm{euc}}=\sqrt{\sum_{i=1}^{m}{\left(x_{i}-y_{i}\right)}^{2}},$$

where *m* indicates the data dimension. With the increase of variables, the distinguishing ability of Euclidean distance becomes worse.

The Manhattan distance is written as [28]

(14) $$d_{\mathrm{man}}=\sum_{i=1}^{m}\left(\right|x_{1i}-y_{1i}\left|+\right|x_{2i}-y_{2i}\left|\right).$$

The Manhattan distance has a fast calculation speed, but when the differences of variables are large, some features will be ignored.

The cosine distance is represented as [28]

(15) $$d_{\cos}=\sum_{i=1}^{m}\left(\frac{x_{1i}y_{1i}+x_{2i}y_{2i}}{\sqrt{x_{1i}^{2}+x_{2i}^{2}}\times \sqrt{y_{1i}^{2}+y_{2i}^{2}}}\right).$$

The cosine distance is suitable for many variables and solving the problems of outliers and sparse data, whereas it discards the useful information contained in the vector length.

The Chebyshev distance is executed as [28]

(16) $$d_{\mathrm{che}}=\max(|x_{i}-y_{i}\left|\right).$$

The Chebyshev distance is generally utilized to calculate the sum of distances, such as the logistic store.

#### 3.1.2. SVM

**Figure 3:**
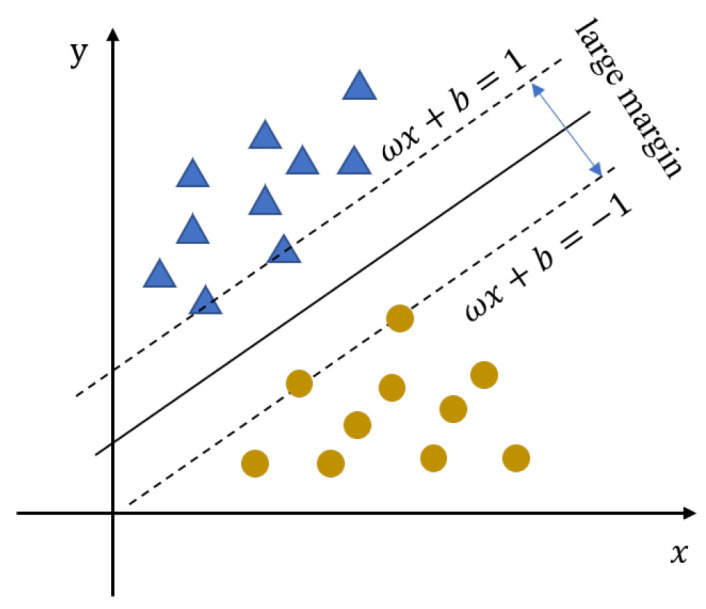
SVM hyperplane for discrimination.

The support vector machine (SVM) is a supervised learning algorithm, which especially supports the binary classification. SVM maps samples into space and finds a hyperplane to maximize the interval between samples. The classification of training samples is divided into two parts, including linear and nonlinear. The linear data could be divided into positive and negative samples [29]. SVM uses a hyperplane to divide the positive and negative samples. The selection of the hyperplane is shown in Figure 3, which can be described as [30]

(17) $$\omega x_{i}+b=0,$$

where ω denotes the normal vector, and *b* indicates the distance between the plane and coordinate origin.

Building an optimized hyperplane in a complex nonlinearly separable problem is done using kernels. The kernel functions are of many types such as Gaussian, polynomial, sigmoid, Cauchy, and so on [31]. Kernel functions map linearly inseparable data to high-dimensional space.

The Gaussian kernel function is performed as [32]

(18) $$k_{\mathrm{gau}}\left(x,y\right)=e^{-\frac{||x-{y||}^{2}}{2\sigma^{2}}},$$

where σ represents the standard deviation. The Gaussian kernel function is commonly used in SVM, and the essence of Gaussian is to map each sample point to an infinite-dimensional feature space, which means the deformation of samples is extremely complex, but the characteristics of each sample are clear.

The polynomial function is denoted by [32]

(19) $$k_{\mathrm{pol}}\left(x,y\right)={\left(x\cdot y+1\right)}^{D},$$

where *D* denotes the degree of the polynomial. The function indicates the similarity of vectors in the training set. The polynomial function is relatively stable, but it involves many parameters.

The sigmoid function is defined as [32]

(20) $$k_{\mathrm{sig}}\left(x,y\right)=tanh\left(x\cdot y+1\right).$$

Sigmoid is an S-shaped function, which is often employed as the activation function of the neural network to map variables between 0 and 1.

The Cauchy function is written as [32]

(21) $$k_{\mathrm{cau}}\left(x,y\right)=\frac{1}{\frac{||x-{y||}^{2}}{\sigma }+1}.$$

The Cauchy function is mainly applied to deal with high-dimensional data.

#### 3.1.3. NB

Naive Bayes (NB) is a discriminant method based on Bayesian theorem and feature condition independence hypothesis [33]. The advantage of NB is that it combines the prior probability and the posterior probability, that is, it avoids the subjective bias of using only the prior probability and the over fitting phenomenon of using sample information alone [34]. However, NB requires few estimated parameters, it is not sensitive to missing data, and the assumption is relatively simple, so the accuracy of the algorithm is affected. According to different assumptions, NB includes Gaussian NB (GNB), Multinomial NB (MNB), Complement NB (CNB), Bernoulli NB (BNB), Categorical NB, and so on [35].

GNB denotes the prior distribution, which is assumed to be Gaussian [36]. BNB is designed for binary discrete data [37]. The Categorical NB assumes that each feature described by the index has its own classification distribution [38]. MNB is utilized to calculate the probability of discrete features [39]. CNB can be used to classify imbalanced datasets when the features do not satisfy the conditions of mutual independence. [40]. NB contains multiple input variables and target variables as model outputs. Let *S* be the state of the variable and $X=(x_{1},x_{2},...,x_{n})$ be the state of *n* input features. To estimate the value of *S* based on *X*, the conditional probability of *S* needs to be calculated by *X*, and the expression is [41]

(22) $$p\left(S|X\right)=\frac{p(X|S)p(S)}{p(X)},$$

where p(S) and p(X) are constants that are obtained from data. p(X|S) can be calculated as [41]

(23) $$p\left(X|S\right)=p\left(x_{1},x_{2},...,x_{n}|S\right)=\prod_{i=1}^{n}p\left(x_{i}|S\right).$$

The expression of p(S|X) can be simplified as [41]

(24) $$p\left(S|X\right)=\frac{p(S)}{p(X)}\prod_{i=1}^{n}p\left(x_{i}|S\right).$$

### 3.2. Ensemble Algorithms

As one of the current research hotspots, ensemble learning has been applied tentatively in many fields, such as image processing, malware detection, and so on [42]. The multi-model properties of ensemble learning enable to avoid the imprecise characteristic of a single model, which also shows potential in solving complex problems.

Ensemble learning refers to strategically generating multiple weak classifiers and then combining them into a strong classifier to complete the discrimination task, which has superior generalization ability. Next, several effective algorithms in some fields will be introduced. The ensemble algorithms are mainly divided into two categories, including serial and parallel algorithms [43]. Random Forest (RF) and Bootstrap Aggregating (Bagging) belong to the parallel algorithms. Boosting and Stacked Generalization (Stacking) are parts of the serial algorithms.

#### 3.2.1. RF

**Figure 4:**
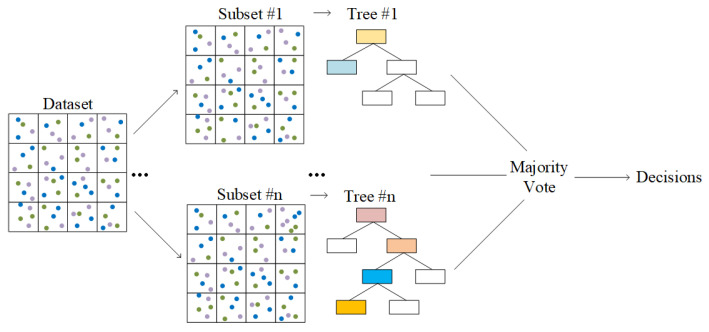
Structure of the random forest classifier.

RF is a classifier containing multiple decision trees, and its output category is determined by the mode of the category output by individual decision trees [44]. The decision tree adopts the top-down recursive method, which constructs a tree with the fastest entropy decline based on information entropy. The information entropy is defined as [45]

(25) $$H=-\sum_{i=1}^{n}p_{i}\ln\left(p_{i}\right),$$

where *H* refers to the information entropy, and *p* indicates the probability. It can be seen from Figure 4 that RF consists of multiple decision trees. Each decision tree will get a discrimination result, and all the results determine the final output. The advantage of RF is that it is able to process high-dimensional data and find the relationship between different variables [46]. The advantage of RF is that it can process high-dimensional data, has strong anti-noise ability, and avoids the overfitting problem.

RF has superior performance in numerous aspects, especially in pathological research and financial investment. However, because of its slow pace, the random forest classifier is not applicable to real-time predictions.

#### 3.2.2. Bagging

**Figure 5:**
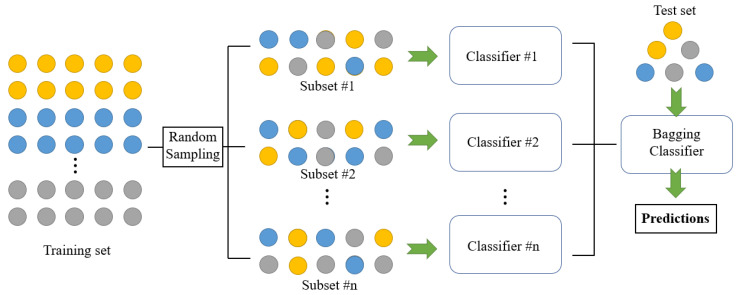
Structure of Bagging classifier.

Bagging is an algorithm framework, which trains several different models respectively, and then lets all models vote to test the output of samples [47]. As shown in Figure 5, Bagging adopts a sampling with replacement to generate multiple training subsets, which are employed to train classifiers [48]. Each training process is independent, so the process could be accelerated by parallel computing [49]. Especially, the training subset of Bagging is randomly selected, which means that different subsets may contain the same data. Moreover, Bagging introduces randomization in the training of each classifier. After training, all classifiers are combined to reduce the variance of prediction results. After the *L*-th iteration, the expectation of the strong classifier is expressed as [50]

(26) $$\phi \left(x\right)=E_{L}\left(x,L\right).$$

The difference between the real value *y* and the predicted value of the weak classifier can be written as [50]

(27) $$E_{L}{\left(y-\phi \left(x,L\right)\right)}^{2}=y^{2}-2yE_{L}\phi \left(x,L\right)+E_{L}\phi^{2}\left(x,L\right).$$

The comparison result of classifiers is described as [50]

(28) $$E_{L}{\left(y-\phi \left(x,L\right)\right)}^{2}\geq {\left(y-\phi \left(x\right)\right)}^{2}.$$

The expectation of multiple weak classifiers is better than that of the strong classifier, that is, Bagging is able to effectively improve the discrimination accuracy, especially when the variance between the variables is large.

#### 3.2.3. Boosting

Similar to Bagging, Boosting also trains multiple weak classifiers to jointly decide the final output [51]. However, weak classifiers are strengthened and trained by weighting in Boosting [52]. Boosting is a framework, which obtains the subset, and utilizes the weak classification algorithm to train to generate a series of base classifiers [53]. The optimization model of Boosting is executed as [53]

(29) $$F_{m}\left(x\right)=F_{m-1}\left(x\right)+{\mathrm{argmin}}_{\theta }\sum_{i=1}^{n}L\left(y_{i},F_{m-1}\left(x_{i}\right)+\theta \left(x_{i}\right)\right),$$

where *L* denotes the greedy optimization. To solve the detailed problem of subsets and classifiers, Boosting derives multifarious algorithms, including Adaptive Boosting (AdaBoost), Gradient Boosting Decision Tree (GBDT), Xtreme Gradient Boosting (XGBoost), and so on.

AdaBoost will select the key classification feature set in the training set for many times. It trains the component weak classifier step by step and selects the best weak classifier with an appropriate threshold. Finally, the best weak classifier for each iteration is selected to construct a strong classifier. However, AdaBoost combines weak classifiers to construct a strong classifier [54]. The weights of each weak classifier are not equal, and the stronger classifier will be assigned the high weight [55]. Specifically, the weighted error of the *k*-th weak classifier $G_{k}\left(x\right)$ is written as [54]

(30) $$e_{k}=P\left(G_{k}\left(x_{i}\right)\neq y_{i}\right)=\sum_{i=1}^{m}w_{ki}I\left(G_{k}\left(x_{i}\right)\neq y_{i}\right),$$

where *w* indicates the output weight. The weight coefficient of the *k*-th $G_{k}\left(x\right)$ is defined as [54]

(31) $$\alpha_{k}=\frac{1}{2}\log\left(\frac{1-e_{k}}{e_{k}}\right).$$

It can be found that the weight coefficient decreases with the increase of weighted error.

The expression of updated weight is [54]

(32) $$w_{k+1,i}=\frac{w_{ki}\exp\left(-\alpha_{k}y_{i}G_{k}\left(x_{i}\right)\right)}{\sum_{i=1}^{m}\exp\left(-\alpha_{k}y_{i}G_{k}\left(x_{i}\right)\right)}.$$

AdaBoost needs a quality dataset because it is hard to handle noisy data and outliers. At present, AdaBoost is being used to classify text and images rather than binary classification problems.

The core of GBDT is that each tree learns the residual of the sum of all previous tree conclusions, which is the accumulation of the real value after adding the predicted value [56]. The fitting error of GBDT, which is replaced by the negative gradient of the loss function, is reduced by multiple iterations [57]. The negative gradient expression of the *i*-th sample in the *t*-th iteration is performed as [56]

(33) $$r_{ti}=-{\left[\frac{\partial L(y_{i},f\left(x_{i}\right))}{\partial f(x_{i})}\right]}_{f\left(x\right)=f_{t-1}\left(x\right)},$$

where $r_{ti}$ denotes the negative gradient, and *L* represents the loss function. After getting the *t*-th decision tree, the optimal solution of the loss function is given by [56]

(34) $$c_{tj}=\arg\min\sum_{x_{i}\in R_{tj}}L\left(y_{i},f_{t-1}\left(x_{i}\right)+c\right),$$

where *c* represents the optimal solution, *R* indicates the region of the child node, and *j* denotes the number of the child node. The optimal solution could be utilized to update the weak classifier [58].

XGBoost adopts a similar theory to GBDT [59]. GBDT applies the first derivative in the loss function, but the loss function of XGBoost is approximated by the second-order Tailor expansion. Furthermore, the objective function of XGBoost imports a regularizer to avoid the over-fitting problem, which is expressed as [60]

(35) $$Obj^{\left(t\right)}=\sum_{i=1}^{n}L\left(y_{i},{\hat{y}}_{i}^{\left(t\right)}\right)+\sum_{i=1}^{t}\Omega \left(f_{i}\right),$$

where $\hat{y}$ denotes the forecasting sample, and Ω represents the regularizer. XGBoost employs regularization to avoid overfitting, and it usually has superior performance in dealing with small and medium datasets.

#### 3.2.4. Stacking

**Figure 6:**
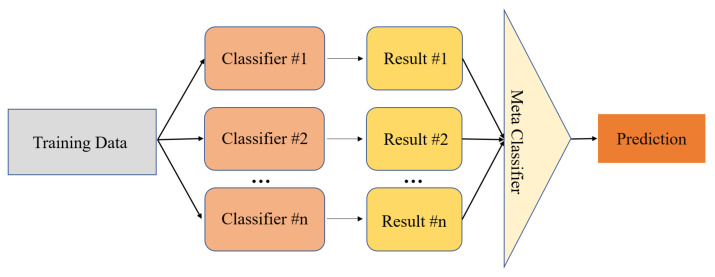
Schematic of a stacking classifier framework.

Stacking is an ensemble technique that combines multiple discrimination results generated by using different learning algorithms on the dataset [61]. Stacking contains two layers of classification models, as shown in Figure 6. The first layer applies various classifiers to predict the result. The result is input into the second layer as the training set. The second layer is utilized to assign higher weights to better classifiers, so the two-layer model could effectively reduce the variance [62]. Hence, Stacking will select several classifiers with good fitting for deciding the final result. However, the good performance of a single classifier does not mean that the combined effect is ideal.

## 4. Interference Discrimination Experiments

In this section, practical sensors-collected remote interference measurement data is employed to analyze the testbed effectiveness. The selected single model algorithms were employed to discriminate the interference of the base station [26]. The selected ensemble algorithms have excellent performance on complex problems. Furthermore, accuracy and recall are applied to assess the performances of algorithms. Accuracy refers to the probability that the models correctly judge the test data, and recall indicates the probability that the models correctly judge the data interfered by the atmospheric duct. As such, the experiments include three parts. (a) Change the size of the dataset; (b) Change the imbalance ratio (IR) of the data size; (c) Test the robustness of algorithms; (d) Time complexity.

### 4.1. Interference Dataset

The dataset is the measurement of the sensor under the TDD system, which is provided by China Mobile Group Jiangsu Co., Ltd. Some base stations were interfered by the atmospheric duct in Jiangsu Province of China, which interfered with the reception of the uplink signal. The data is collected from 240,000 antennas in Jiangsu, including the longitude, latitude, time, antenna height, and down tilt angle.

**Figure 7:**
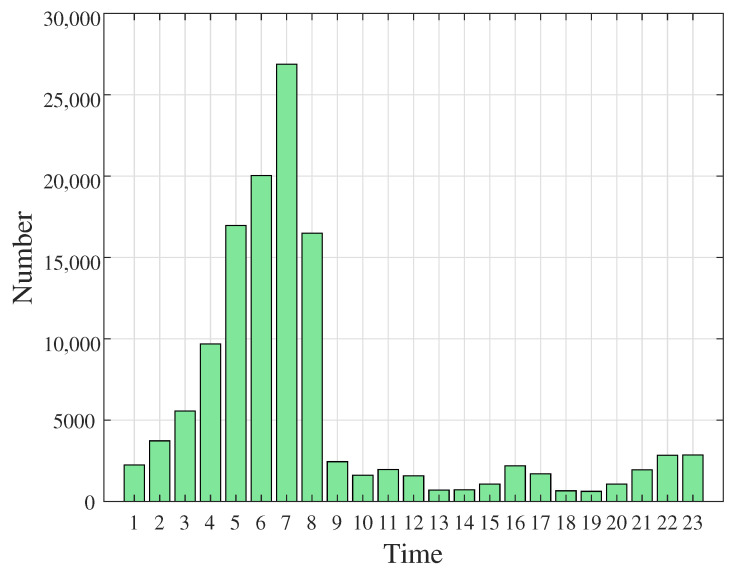
Number of interfered base stations.

Figure 7 shows the number of interfered base stations, which gradually increases from 1.00 a.m. to 7.00 a.m., with the number dropping dramatically from 8.00 a.m. The trend shows that the atmospheric duct usually appears from midnight to the morning. From the explanation of meteorology, the temperature of the ground drops quickly and the lower atmosphere is prone to temperature inversion from midnight to the morning, which means that within a certain height, the temperature increases with the vertical height, which causes the atmospheric duct phenomenon.

The meteorological data is obtained from CFSv2, which is a fully coupled model representing the interaction between the earth’s atmosphere, oceans, land, and sea ice [63]. The meteorology of CFSv2 includes the temperature, relative humidity, pressure, salinity, and so on. We download the temperature, relative humidity, and pressure data, which is related to the atmospheric duct, to match with the base station according to the longitude and latitude.

### 4.2. Algorithm Settings

The hardware and software configurations of experiments are listed in Table 1. The algorithms in Section 3 are selected to test the performance of the interfered dataset. Unless otherwise specified, all parameters are set to the values in Table 2 by default. The empirical results show that a large proportion of algorithms converge after 100 iterations, which is chosen as the maximum number of iterations in our experiments. Particularly, the iterations of AdaBoost are 500 because the higher iterations of the algorithm will significantly improve the discrimination results.

### 4.3. Sensitivity of the Algorithms to the Data Size

To verify the influence of different data sizes, the size of the training set is set as 20,000, 30,000, 40,000, 50,000, and 60,000, respectively. The IR of each training set is 5:1: for instance, in the training set of 20,000, about 3333 pieces of data are interfered by the atmospheric duct, and the rest are normal. Moreover, the equivalent data is sampled per hour to form the training set.

The size of the test set is set to 20% of the total number of the training set. The number of the interfered data and the normal data are the same in the test set, which is applied to emphasize the learning ability of the algorithms for the imbalanced dataset. Similarly, the equivalent data is sampled per hour to form the test set, which ensures fairness in the time domain.

There is no overlap between the training set and the test set. When the size of the training set changes, both the training set and the test set will be selected randomly. Besides, two indicators, including accuracy and recall, are applied to evaluate the learning ability of the algorithms. The expression of accuracy can be expressed as

(36) $$Acc=\frac{TP+TN}{TP+TN+FP+FN},$$

where TP is the true positive, TN is the true negative, FP is the false positive, and FN is the false negative. In the interference discrimination problem, TP refers to the interfered samples that are judged correctly by algorithms, TN denotes the interfered samples that are judged incorrectly, FP represents the undisturbed samples that are judged correctly, and FN indicates the undisturbed samples that are judged incorrectly.

The expression of recall is defined as

(37) $$Recall=\frac{TP}{TP+FN}.$$

The recall is utilized to reflect the judgment ability of the algorithm for specific indicators, which is especially adopted to display the judgment of the interfered data in the interference discrimination problem.

**Figure 8:**
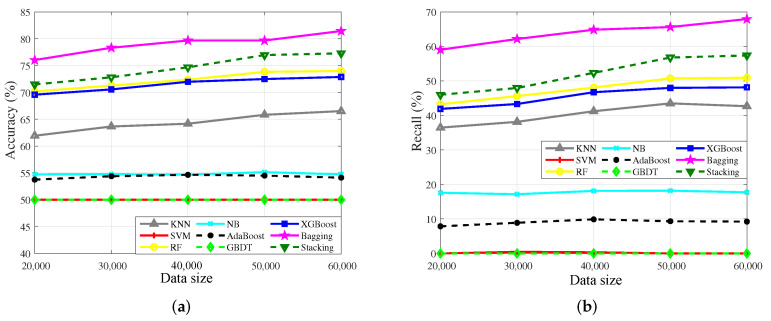
Accuracy and recall results of single model algorithms and ensemble algorithms with different data sizes. (**a**) Accuracy results. (**b**) Recall results.

Table 3 shows the specific classification results on different datasets. The accuracy results of single model algorithms and ensemble algorithms are illustrated in Figure 8a, and the recall of two kinds of algorithms is shown in Figure 8b.

In Figure 8a, the accuracy of all algorithms keeps improving with the increase of data, which means that the amount of data has a significant impact on the accuracy. Specifically, Bagging has the highest accuracy, which demonstrates it could better characterize the complex nonlinear relationship between variables. The recall has a similar trend with the accuracy, as shown in Figure 8b, which shows that the recall of Bagging is higher than the others, that is, Bagging could well learn the characteristics of the minority in the imbalanced datasets.

Stacking, RF, and XGBoost have stationary performance on the dataset, which validates that the three algorithms could fit the complex nonlinear relationship among variables well. The accuracy of kNN is generally precise, which indicates that there are a few differences among the variables, so distance matching is hard to find the internal relationship among variables. NB only needs a few samples to achieve high accuracy, so the accuracy has changed rarely when the amount of data is sufficient. Meanwhile, the generalization ability of the model is weak, so the learning ability of the minority is poor. The accuracy of AdaBoost is not high, because the weights tend to the classifiers that have superior performance, and the generalization ability of the model is affected.

However, the accuracy results of SVM and GBDT only attain 50.00%, and the recall results of the two algorithms are almost 0.00%. It is revealed that the two algorithms judge the data as normal data with a high proportion in the training set. We also test the ideal case with a 1:1 imbalance ratio. The experimental results show that the accuracy and recall of the two algorithms have improved significantly, which indicates that the model training of the two algorithms tends to characterize the data features with a high proportion, that is, SVM and GBDT are not sensitive to the minority.

The accuracy of partial algorithms decreases when the data is increasing because the selection of the datasets is random. Besides, with the increase of data, the weight of learning will change, which also affects the accuracy.

Basically, the performance of ensemble algorithms generally outperforms single model algorithms in the interference discrimination problem, which indicates that ensemble algorithms are available for characterizing complex nonlinear relationships. Besides, the accuracy of partial algorithms decreases when the data is increasing because the selection of datasets is random. Besides, with the increase of data, the weight of learning will change, which also affects the accuracy.

### 4.4. Sensitivity of the Algorithms to IR

Typically, IR refers to the ratio of the majority to the minority in the training set. In this paper, IR represents the ratio of undisturbed samples to interfered samples in the training set. To verify the influence of IR on algorithms, the IR of the training set is set as 3:1, 5:1, 7:1, 9:1, and 11:1, respectively. The size of all training sets is 40,000. Meanwhile, the equivalent data is sampled per hour to form each training set.

As mentioned, the size of the test set is set to 20% of the number of the corresponding training set. The number of the interfered data and the normal data are the same in each test set. The equivalent data is sampled per hour to form the test set. Besides, there is no intersection between the training set and the test set, and the dataset is selected randomly. Similarly, accuracy and recall are applied to evaluate the algorithms.

**Figure 9:**
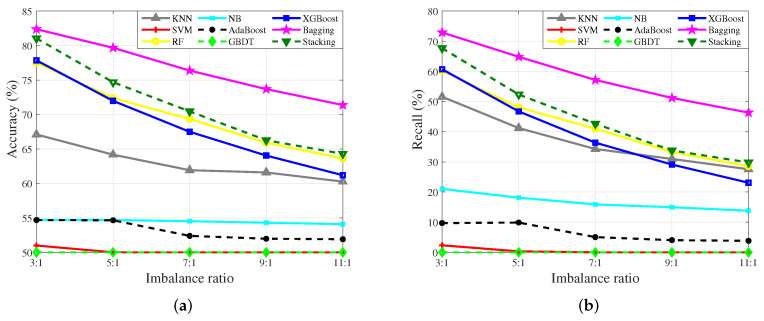
Accuracy and recall results of single model algorithms and ensemble algorithms with different IR. (**a**) Accuracy results. (**b**) Recall results.

The impact of IR on the algorithms is listed in Table 4. The accuracy results of single classification algorithms and ensemble algorithms are shown in Figure 9a. The recall results of two kinds of algorithms are shown in Figure 9b.

It is shown in Figure 9a that with the increase of the IR, the accuracy results of all algorithms decrease by degrees, which means that IR has an appreciable effect on the algorithms. When the IR is 3:1, the results among Bagging, Stacking, XGBoost, and RF are close. It means that when the value of IR is small, the ensemble algorithms are capable of achieving comparatively thorough learning of the dataset. However, with the increase of IR, the decline range of Bagging is smaller than the others, which validates that Bagging is able to learn the highly imbalanced dataset well.

With the increase of the IR, the accuracy results of Stacking, XGBoost, and RF are dropping obviously. When the IR is 11:1, the results of the three algorithms are close to the result of kNN. Moreover, similar results could be found in Figure 9b. The recall of kNN is even higher than that of XGBoost. It is reasonable that IR has a great impact on the ensemble algorithms, that is, the characteristics of the minority in highly imbalanced datasets are difficult to learn. Meanwhile, the reduction of the minority means that the characteristics of the minority will be more prominent, so kNN is easy to match the point at this time.

As mentioned before, NB is driven by a few samples, so the performance of NB changes little. The performance of AdaBoost is still not improved on the imbalanced dataset due to the weight distribution problem.

**Figure 10:**
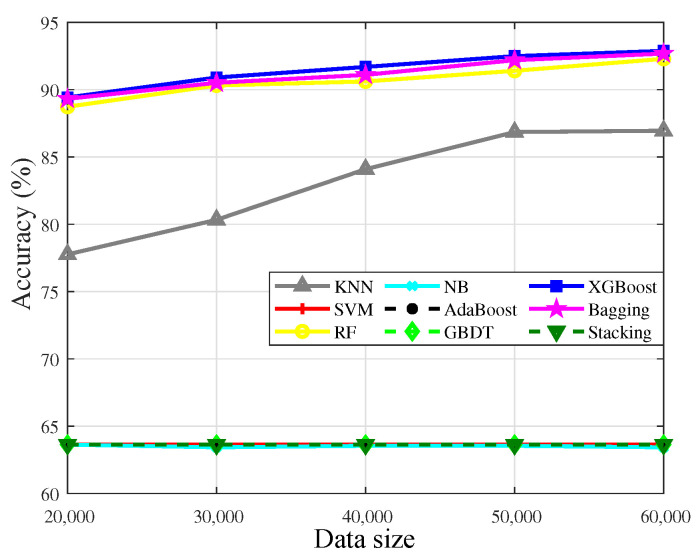
Accuracy results of algorithms in the training set that contains 1% abnormal data.

**Figure 11:**
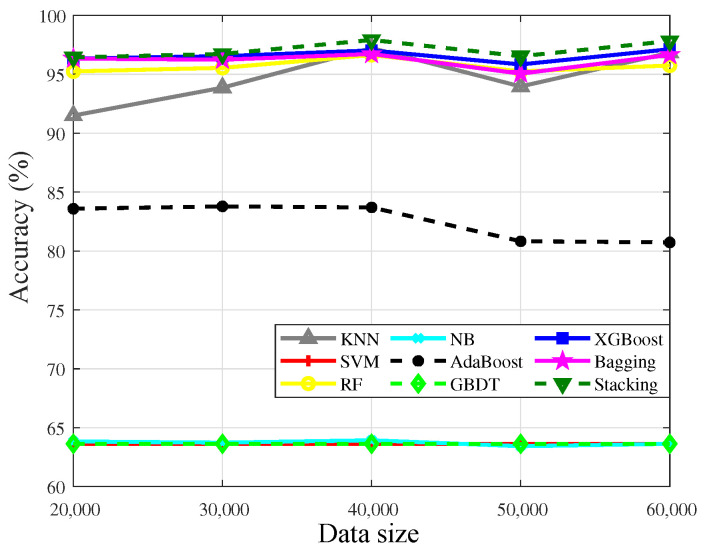
Accuracy results of algorithms in the training set that contains 5% abnormal data.

From the experimental results illustrated in Figure 10 and Figure 11, SVM and GBDT are not sensitive to the minority. However, it is observed that when the IR is 3:1, the accuracy of SVM is 50.99% and the recall of SVM is 2.33%. It means that SVM is able to be utilized to characterize the minority only when the IR is low enough, which further confirms that the learning ability of SVM for the imbalanced dataset is weak.

### 4.5. Robustness Analysis of the Algorithms

Data measurement failure caused by equipment power failure is unavoidable. In consequence, the abnormal data is included in our dataset considering the actual equipment conditions. The main forms of the abnormal data are the down tilt angle, equaling −$1^{\circ }$, when the antenna height is 0, and so forth. Some abnormal data is added to the training set to analyze the robustness of the algorithms.

We adopt the training set of Part C as the initial training set of the experiment. The IR of the training set is still 5:1. In the following, the abnormal data randomly replaces the same amount of data in the training set, and the replaced proportion is 1% and 5% of the training set, respectively.

The test set does not change in all experiments. About 1000 pieces of abnormal data are employed to form the test set. The equivalent abnormal data is sampled per hour to form the test set. In addition, there is no overlap between the training set and the test set. The accuracy is used for evaluating the robustness of the algorithms.

Table 5 shows the learning ability of the algorithms for abnormal data. The accuracy results of algorithms, which are trained by the 1% dataset, are shown in Figure 10. It can be seen that with the increase of the training data, the accuracy results of most algorithms are improving. The accuracy of XGBoost is higher than the others, which means that XGBoost could learn the characteristics of abnormal data well even if the number of data is small. Moreover, the performance of RF, kNN, and Bagging is also stationary.

The accuracy results of SVM, AdaBoost, NB, and Stacking are 63.63% when the training set contains 1% abnormal data. By analyzing the test set, we find that the data, which is not affected by the atmospheric duct, accounts for 63.63% of the training set. It means that the above four algorithms are not sensitive to samples when the number of samples is extremely low.

Figure 11 presents the robustness of the algorithms on the training set with 5% abnormal data. It is observed that the increase of the abnormal data from 1% to 5% improves the accuracy of the algorithms. Stacking outperforms other algorithms. In Figure 11, 40,000 pieces of training data achieve higher accuracy than that of 50,000 pieces, which indicates that the data characteristics contained in the randomly selected database have not been well learned by the algorithms. The accuracy difference between 40,000 and 50,000 data is about 1%, which indicates that the random data selection will cause fluctuations, but there is no large deviation,.

Compared to Figure 10 and Figure 11, it can be known that the increase of the abnormal data from 1% to 5% greatly improves the accuracy of kNN and Stacking, which means the two algorithms will be trained well when the number of the abnormal data reaches a certain level, but it also reflects that they are not sensitive to a few samples in a highly imbalanced dataset.

Moreover, AdaBoost is also greatly affected by the number of abnormal data, although the accuracy is not ideal. However, the increase of the abnormal data does not improve the accuracy of SVM and GBDT, which means the learning ability of the two algorithms is weak when the dataset is a highly imbalanced set and there are complex nonlinear relationships between the variables. Besides, with the increase of the abnormal data, the accuracy of NB changes slightly, which means that NB is sensitive to the abnormal data, that is, NB has ordinary learning ability for the highly imbalanced dataset.

### 4.6. Time Complexity

To analyze the algorithm efficiency, we list the time complexity of each algorithm, namely, the floating-point operations. To ensure comparison consistency, the time complexity is the result of running the code once in each algorithm. The time complexity and order of the algorithms are listed in Table 6 where *n* represents the number of inputs.

The time complexity is explained in detail. *k* denotes the dimension of a single sample characteristics. *c* indicates the number of categories. *m* represents the number of decision trees. *d* refers to the depth of the tree. ${\left||x|\right|}_{0}$ means all non missing items in the training data. The order of Bagging and Stacking is related to the time complexity of base classifiers.

Specifically, the order of SVM is quadratic, which is unfriendly to the problem with considerable training data. The order of Bagging and Stacking depends on the selected base classifier, that is, when the order of the base classifier is low, the time complexity of Bagging and Stacking is acceptable. XGBoost adopts fractional data block parallelism, which enables the time complexity competitive.

To intuitively compare the complexity of the algorithm, we run the program in the configuration environment of Part B, and listed the test time in Table 6. Without loss of generality, each algorithm only compares the training time. The training set is selected from Part C, the data size is 40,000, and the IR is 5:1.

The time consumption of algorithms is shown in Table 6. It can be found that although the order of ensemble algorithms is generally higher than that of single model algorithms, its time consumption in solving the complex interference discrimination is still acceptable.

## 5. Conclusions

In this paper, a remote interference discrimination testbed with several promising AI algorithms was proposed to assist operators in identifying interference. The introduced framework for the testbed and the detailed design of the modules were presented. Furthermore, the testbed with 5,520,000 network-side data made a consistent comparison of nine AI algorithms. Numerical results illustrated that the ensemble algorithm had higher interference discrimination accuracy than the single model algorithm. Operators could select the algorithm with appropriate complexity to discriminate interference according to the conditions of hardware equipment. Considering the fluctuating accuracy of the algorithm, future work will consider optimizing the ability of the algorithm to learn data characteristics so that the algorithm can achieve stable performance. Moreover, the accuracy upper bound of remote interference discrimination deserves further exploration.

## Figures and Tables

**Table 1:** Hardware and software configurations.

Designation	Configuration
Core	i5-4210 H 2.90 GHz
Operating system	Windows 10
Random-access memory	12.0 GB
Python	3.7
Tensorflow	2.0.0

**Table 2:** Settings of the key parameters in the algorithms.

Category	Algorithms		Parameters	Value
Single model algorithms	kNN		Number of neighbors	1
	SVM		Kernel	Linear/Radial basis function
			Maximum number of iterations	100
	NB		Type	Gaussian/Bernoulli/Complement
Ensemble algorithms	RF		Number of trees in the forest	100
	Bagging		Number of base estimators in the ensemble	100
	Boosting	AdaBoost	Maximum number of estimators	500
			Learning rate	0.01
		GBDT	Number of boosting stages to perform	100
			Learning rate	0.01
		XGBoost	Number of decision trees	100
			Learning rate	0.1
	Stacking		Estimators	Lr/rf/kNN/cart/svc/bayes
			Final estimator	LogisticRegression

**Table 3:** Results of changing the data size of the algorithms.

IR			5:1									
**Size of Training Data**			**20,000**		**30,000**		**40,000**		**50,000**		**60,000**	
**Indicators**			**Acc**	**Recall**	**Acc**	**Recall**	**Acc**	**Recall**	**Acc**	**Recall**	**Acc**	**Recall**
Single model algorithms	kNN		61.93	36.46	63.65	38.14	64.17	41.21	65.83	43.49	65.52	42.67
	SVM		50.00	0.00	50.02	0.46	50.01	0.33	50.00	0.00	50.00	0.00
	NB		54.74	17.58	54.76	17.16	54.69	18.13	55.14	18.19	54.74	17.73
Ensemble algorithms	RF		70.18	43.23	71.27	45.61	72.38	48.07	73.83	50.73	74.00	50.92
	Bagging		**76.03**	**59.04**	**78.32**	**62.15**	**79.67**	**64.84**	**79.67**	**65.61**	**81.44**	**67.93**
	Boosting	AdaBoost	53.75	7.85	54.38	8.89	54.65	9.88	54.50	9.33	54.12	9.23
		GBDT	50.00	0.00	50.00	0.00	50.00	0.00	50.00	0.00	50.00	0.00
		XGBoost	69.58	41.89	70.56	43.31	71.98	46.73	72.49	47.98	72.88	48.14
	Stacking		71.49	45.99	72.82	47.95	74.68	52.34	76.96	56.78	77.28	57.38

**Table 4:** Results of changing the IR on the algorithms.

Size of Training Data			40,000									
**IR**			**3:1**		**5:1**		**7:1**		**9:1**		**11:1**	
**Indicators**			**Acc**	**Recall**	**Acc**	**Recall**	**Acc**	**Recall**	**Acc**	**Recall**	**Acc**	**Recall**
Single model algorithms	kNN		67.10	51.57	64.17	41.21	61.92	34.26	61.59	30.98	60.26	27.55
	SVM		50.99	2.33	50.01	0.33	50.00	0.00	50.00	0.00	50.00	0.00
	NB		54.71	21.04	54.69	18.13	54.52	15.90	54.30	14.96	54.09	13.81
Ensemble algorithms	RF		77.51	60.24	72.38	48.07	69.35	40.99	65.91	33.29	63.61	28.59
	Bagging		**82.37**	**72.89**	**79.67**	**64.84**	**76.37**	**57.16**	**73.68**	**51.20**	**71.37**	**46.33**
	Boosting	AdaBoost	54.68	9.71	54.65	9.88	52.39	5.06	51.98	4.04	51.90	3.82
		GBDT	50.00	0.00	50.00	0.00	50.00	0.00	50.00	0.00	50.00	0.00
		XGBoost	77.85	60.69	71.98	46.73	67.49	36.37	64.04	29.11	61.19	23.08
	Stacking		81.04	67.70	74.68	52.34	70.45	42.63	66.26	33.79	64.29	29.81

**Table 5:** Results of the algorithms on abnormal data.

Indicator			Accuracy									
**Size of Training Data**			**20,000**		**30,000**		**40,000**		**50,000**		**60,000**	
**Proportion of Abnormal Data**			**1**%	**5**%	**1**%	**5**%	**1**%	**5**%	**1**%	**5**%	**1**%	**5**%
Single model algorithms	kNN		77.76	91.50	80.33	93.87	84.09	97.23	86.85	93.97	86.95	96.83
	SVM		63.63	63.63	63.63	63.63	63.63	63.63	63.63	63.63	63.63	63.63
	NB		63.63	63.83	63.43	63.73	63.54	63.93	63.54	63.43	63.43	63.63
Ensemble algorithms	RF		88.73	95.25	90.31	95.55	90.61	96.64	91.40	95.25	92.29	95.75
	Bagging		89.32	96.34	90.51	96.24	91.10	96.73	92.19	95.06	92.68	96.65
	Boosting	AdaBoost	63.63	83.59	63.63	83.79	63.63	83.70	63.63	80.83	63.63	80.73
		GBDT	63.63	63.63	63.63	63.63	63.63	63.63	63.63	63.63	63.63	63.63
		XGBoost	**89.42**	96.34	**90.90**	96.54	**91.69**	97.03	**92.49**	95.84	**92.88**	97.13
	Stacking		63.63	**96.44**	63.63	**96.73**	63.63	**97.92**	63.63	**96.54**	63.63	**97.82**

**Table 6:** Time complexity of the algorithms.

Algorithms	Time Complexity	Order	Test
kNN	O(kn)	O(n)	1.49 s
SVM	$O(n^{2})$	$O(n^{2})$	8.20 s
NB	O(ckn)	O(n)	1.02 s
RF	O(kdmnlogn)	O(nlogn)	3.98 s
Bagging	O(Base)	O(Base)	11.52 s
AdaBoost	O(knlogn)	O(nlogn)	3.49 s
GBDT	O(kdnlogn)	O(nlogn)	2.46 s
XGBoost	$O(md||x|{|}_{0}+||x|{|}_{0}logn)$	O(logn)	1.79 s
Stacking	O(Base)	O(Base)	47.78 s

## Data Availability

The data that support the findings of this study are available from the corresponding author upon reasonable request.

## Abbreviations

The following abbreviations are used in this manuscript:

IoT	Internet of Things
TDD	Time-Division Duplex
GP	Guard Period
kNN	k-Nearest Neighbors
SVM	Support Vector Machine
NB	Naive Bayes
RF	Random Forest
AdaBoost	Adaptive Boosting
GBDT	Gradient Boosting Decision Tree
XGBoost	Xtreme Gradient Boosting
Bagging	Bootstrap Aggregating
Stacking	Stacked Generalization
PE	Parabolic Equation
IR	Imbalance Ratio
